# Relationship Between EGFR Mutation Status and Clinicopathological Parameters in Non-Small Cell Lung Carcinomas and Correlation of PD-L1 Expression

**DOI:** 10.3390/medicina62081521

**Published:** 2026-08-07

**Authors:** Sumru Cagaptay, Anil Aysal Agalar, Kubra Canaslan, Duygu Gurel, Buket Timur, Emine Cagnur Ulukus, Fatma Ilknur Ulugun, Volkan Karacam, Ilhan Oztop

**Affiliations:** 1 Department of Medical Pathology, Faculty of Medicine, Dokuz Eylul University, Izmir 35330, Turkey; 2 Department of Medical Oncology, Faculty of Medicine, Dokuz Eylul University, Izmir 35330, Turkey; 3 Department of Thoracic Surgery, Faculty of Medicine, Dokuz Eylul University, Izmir 35330, Turkey

**Keywords:** EGFR, PD-L1, driver mutations, histopathological features, clinical features

## Abstract

*Background and Objectives:* In this study, we investigate clinical and pathological parameters that may be associated with EGFR mutations. We aimed to contribute to the literature by understanding other potential clinical and pathological factors that could be useful in predicting patients with a high probability of responding to immunotherapy and by investigating the relationship between PD-L1 expression and driver mutations. *Materials and Methods:* This study included 141 biopsy specimens and 97 resection specimens diagnosed with NSCLC between 2015 and 2022. Statistical analyses were performed using the IBM SPSS Version 29.0 program. *Results:* In our study, consistent with the literature, a significant association was found between the presence of EGFR mutations and female sex and non-smoking status (*p* < 0.001). No statistically significant association was found between PD-L1 expression status and individual driver alterations, including EGFR, BRAF, ALK, and ROS1 (*p* > 0.05 for all). The association between PD-L1 expression status and non-adenocarcinoma histological types, particularly squamous cell carcinoma (10.1%) and NSCLC-NOS (14.5%), was statistically significant; PD-L1 positivity was higher in non-adenocarcinoma tumors (70.6%) compared to adenocarcinomas (43.3%; *p* = 0.009). *Conclusions: * In groups of patients with early-stage and locally advanced disease, the mean overall survival time was significantly lower for those with ALK translocations, which contrasts with the existing literature, and this finding was determined to be an independent poor prognostic factor (HR: 8.6, 95% CI: 1.9–38, *p* = 0.004).

## 1. Introduction

Lung cancer is the leading cause of cancer-related death [1]. Non-small cell lung cancer (NSCLC) is the most common type of lung cancer. Most NSCLC patients are diagnosed at an advanced stage and are thus inoperable. In these patients, targeted therapies guided by molecular diagnostics have become the standard of care, specifically for oncogene-addicted tumors [2]. Currently, the most widely used predictive biomarkers are activating mutations in EGFR, KRAS, and BRAF, alongside ALK and ROS1 rearrangements. EGFR gene mutations occur in approximately 10% to 15% of lung adenocarcinomas in Western populations, whereas higher frequencies ranging from 30% to 50% are reported in Asian cohorts, with intermediate rates (18–24%) routinely documented in Turkish cohorts [3,4,5]. The most common classic alterations account for 85% to 90% of EGFR mutations, comprising a diverse spectrum of in-frame exon 19 deletion variants (most commonly E746_A750del), in addition to the exon 21 L858R point mutation. These two mutations account for 85% to 90% of EGFR mutations [2]. Previous studies have reported different histopathological and prognostic features between EGFR mutant-type and EGFR wild-type patient groups in lung adenocarcinomas [6,7,8]. Furthermore, lesions with exon 19 deletions and exon 21 point mutations exhibit different clinical behaviors, and previous research has shown that lesions with exon 19 mutations respond better to tyrosine kinase inhibitors (TKIs) and platinum-based chemotherapy than lesions with exon 21 mutations in a population with unresectable tumors [9,10,11].

In patients with poor performance status, a biopsy is performed on the most accessible lesion. However, due to tumor heterogeneity, a single sample may not be sufficient to accurately represent the mutation status. Therefore, the question arises as to what other factors may be associated with EGFR mutations. Our study aims to investigate potential clinical and histopathological parameters that may be associated with EGFR mutation and to provide data to inform the selection of appropriate treatment protocols for these patients. Current guidelines recommend immunotherapy for patients without driver mutations or with unknown mutation status [12]. Many studies have appeared in the literature showing that anti-programmed cell death receptor-1 (PD1) and anti-programmed cell death ligand-1 (PD-L1) agents are highly effective against NSCLC and that immunotherapy significantly prolongs overall survival compared to standard chemotherapy [13,14].

Great efforts are being undertaken to identify predictive markers for medication responsiveness to the PD-1/PD-L1 immune checkpoint in patients with poor prognosis [15,16].

The most suitable patient candidates for immunotherapy are determined via the immunohistochemical detection of PD-L1, which is expressed particularly in the cell membrane and cytoplasm [12].

Patients with PD-L1 immunoexpression have been shown to benefit significantly from immunotherapeutics [6,14]. Prior research indicates that PD-L1 positivity in tumor cells correlates with elevated somatic mutations marked by neoantigen expression and that immunotherapy demonstrates greater efficacy in malignancies exhibiting a high mutation rate. Although there are numerous studies investigating the relationship between PD-L1 expression and driver mutation status, the results are contradictory and show heterogeneity [17,18].

In our study, we investigate clinical and pathological parameters that may be associated with EGFR mutations. We also aim to contribute to the literature by elucidating other potential clinical and pathological factors that may be useful in predicting patients with a high likelihood of responding to immunotherapy, as well as the relationship between PD-L1 expression and driver mutations.

## 2. Materials and Methods

### 2.1. Patient Selection and Data Collection

Patient records between 2015 and 2022 were retrospectively examined from the hospital registry’s electronic archives. Within our department, patients diagnosed with NSCLC have undergone molecular analyses of EGFR, ALK, and ROS since 2015 and PD-L1 immunohistochemical analysis since 2019, in accordance with clinical requests.

In our department, patient selection criteria for molecular testing were determined in accordance with both national regulations (Turkish Thoracic Society) and international consensus guidelines, including ESMO (European Society of Oncology Guidelines) and the International Joint Guidelines (IASLC/AMP/CAP). Accordingly, all patients diagnosed with advanced (Stage IIIB–IV) or recurrent non-small cell lung cancer (NSCLC) (especially including adenocarcinoma, adenosquamous carcinoma, and morphologically undifferentiated NSCLC), regardless of their smoking history, were directly referred to a comprehensive molecular testing panel (including EGFR, ALK, and ROS1 analysis). In contrast, molecular testing was not routinely performed in patients diagnosed with squamous cell carcinoma but was conducted only upon specific clinical request in younger patients (under 50 years of age) or those who had never smoked or had low smoking exposure [19,20,21].

Cases diagnosed with NSCLC based on resection specimens (*n* = 97) and subjected to at least one molecular analysis for EGFR, ALK, or ROS1 and cases diagnosed based on biopsy specimens (*n* = 141) and subjected to PD-L1 immunohistochemical analysis in addition to at least one molecular test were consecutively included in this study.

We excluded cases in which we could not assess the outcomes of molecular and immunohistochemical analyses and for which histopathological and clinical data were unavailable. Data regarding the patients involved in this study—encompassing age, sex, date of diagnosis, mortality status, and date of death—were retrieved from the hospital registry system. Clinical information, such as clinical stage, recurrence and metastasis status, dates, treatment type, and smoking history, was provided by the oncology department of our hospital.

### 2.2. Histopathological Evaluation

**Figure 1:**
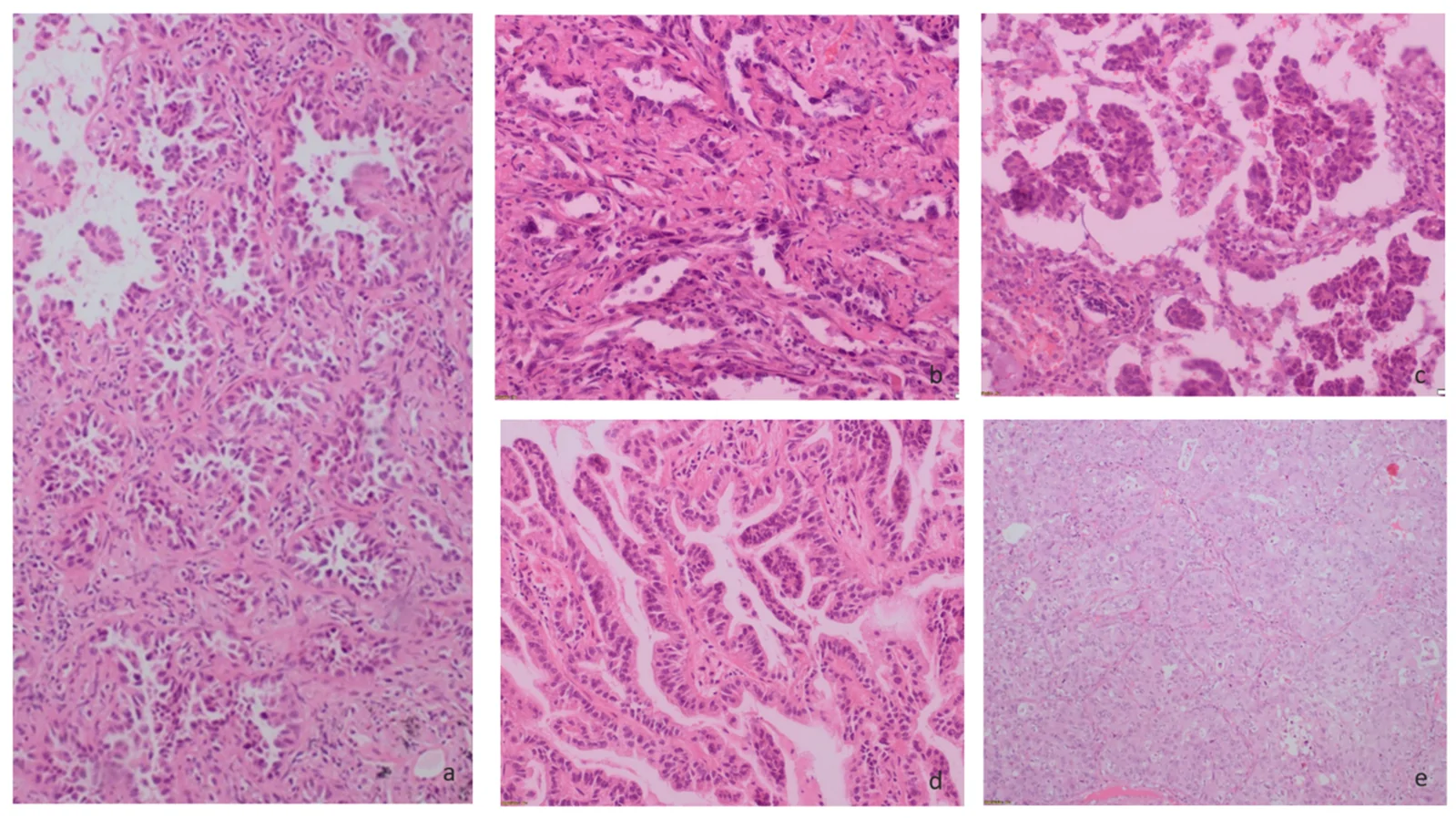
Invasive non-mucinous adenocarcinoma patterns (H&E X200): (**a**). lepidic pattern; (**b**). acinar pattern; (**c**). micropapillary pattern; (**d**). papillary pattern; (**e**). solid pattern.

Histopathological parameters—such as histological subtype, grade, predominant histological pattern (Figure 1), surgical margin status, tumor grade, lymph node metastasis, number of metastatic lymph nodes, lymphovascular space invasion, perineural invasion, pleural invasion, pTNM stage, and immunohistochemical TTF-1 and Napsin-A expression status—were obtained retrospectively from pathology reports.

### 2.3. Immunohistochemical Examination

**Figure 2:**
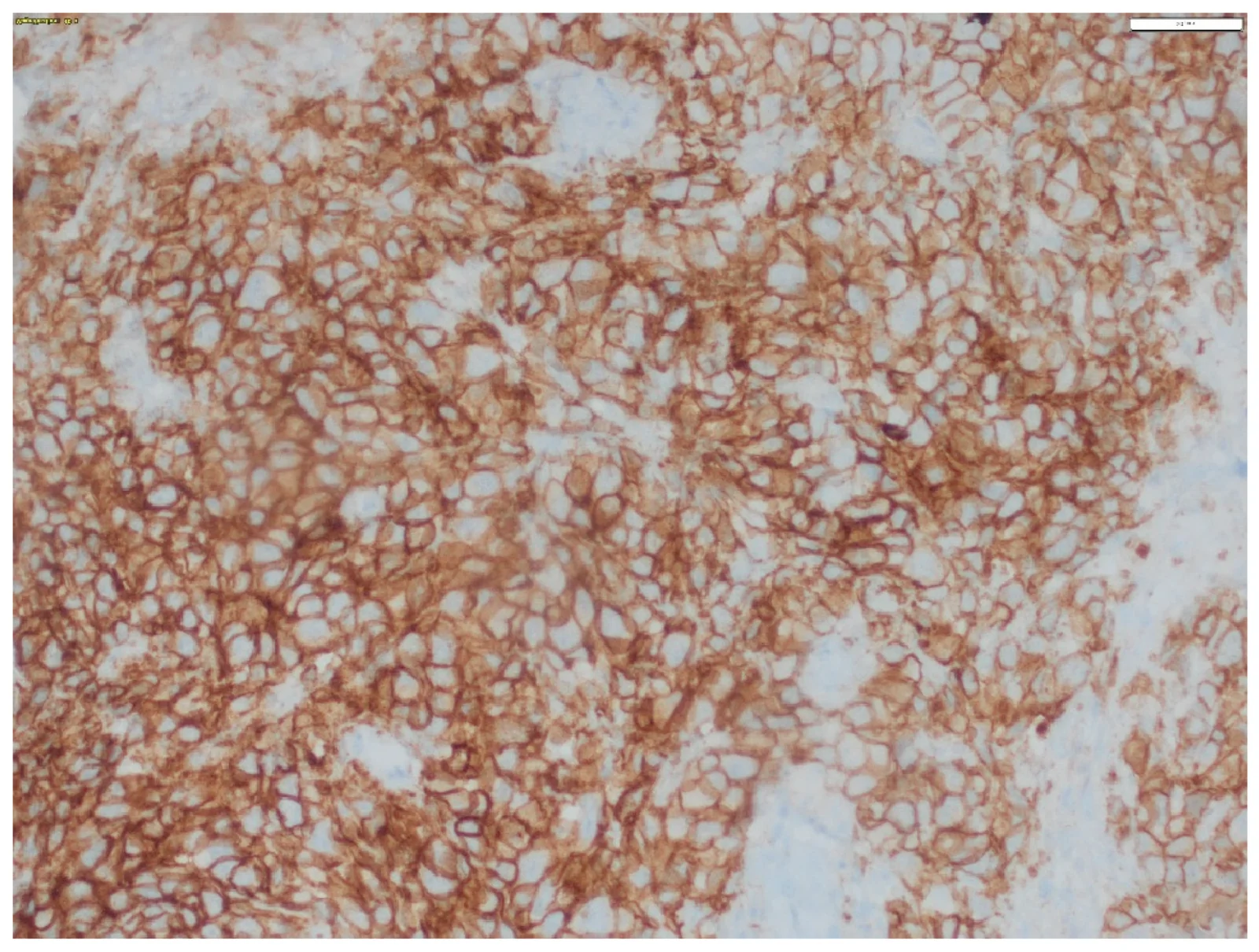
Membranous staining pattern with PD-L1 immunohistochemistry (IHC, original magnification ×10).

Immunohistochemical analyses were performed using a Ventana Benchmark ULTRA (Ventana Medical Systems, Tucson, AZ, USA) fully automated staining device. TTF-1 monoclonal antibodies (clone SP141) and Napsin-A monoclonal antibodies (clone MRQ-60) were used. PD-L1 immunohistochemical analysis was performed using the PD-L1 monoclonal antibody (clone SP263). Membrane staining (Figure 2) of any intensity was considered positive, and the tumor proportion scoring (TPS) value was calculated based on the number of positively stained cells per 100 viable tumor cells. Background staining, staining in cells other than tumor cells, diffuse cytoplasmic staining in tumor cells, necrosis, and staining in areas of intense hemorrhage were not considered in calculating the score. A TPS value of less than 1% was considered negative, and 1% and above was considered positive. Staining percentages were categorized into three groups: less than 1%, 1–49%, and 50% and above.

### 2.4. Molecular Analyses

Molecular analysis results were obtained retrospectively from pathology reports.

#### 2.4.1. EGFR and BRAF Mutation Analysis and Evaluation

To ensure the analytical validity of molecular assays, all formalin-fixed paraffin-embedded (FFPE) tissue sections underwent rigorous pathological examination prior to DNA extraction. A minimum tumor cell density of more than 20% was required for both assay platforms; samples below this threshold were subjected to manual macrodissection on unstained slides to enrich the tumor cell population. The EGFR mutation status of exons 18, 19, 20, and 21, as well as BRAF mutation status (specifically targeting exon 15), was assessed using two complementary methodologies, subject to laboratory availability throughout the study period. Real-time PCR analysis was performed using the Cobas^®^ EGFR Mutation Assay (Roche Diagnostics, Indianapolis, IN, USA) and the Cobas^®^ BRAF V600 Mutation Assay (Roche Diagnostics, Indianapolis, IN, USA), which have a limit of detection (LOD)/variant allele frequency (VAF) threshold of approximately 1% to 5% depending on the specific mutation type. Alternatively, pyrosequencing was performed using the Therascreen^®^ EGFR Pyro Kit and the Therascreen^®^ BRAF Pyro Kit in the Q24^®^ automated system (Qiagen, Hilden, Germany). The LOD/VAF limit for detecting low-frequency variants is approximately 5%.

#### 2.4.2. ALK and ROS-1 Translocation Analysis and Evaluation

ALK and ROS1 translocation analyses were performed via fluorescence in situ hybridization (FISH) using the probes “Zytolight SPEC ALK Break Apart FISH (Zytovision, Bremerhaven, Germany)” and “Zytolight SPEC ROS-1 Break Apart FISH (Zytovision, Germany)”. ALK and ROS-1 FISH analysis and evaluation were performed in accordance with the evaluation criteria of the “IASLC Atlas of Molecular Testing for Targeted Therapy in Lung Cancer”; break-apart and isolated 3′ signal states on at least 50 tumor cells were considered positive when seen at a threshold value of 15% and above [22].

### 2.5. Statistical Analyses

Statistical analyses were performed using IBM SPSS Statistics for Windows, version 29.0 (IBM Corp., Armonk, NY, USA). The chi-square and Fisher’s exact tests were used to compare categorical data. The independent samples *t*-test was employed to compare normally distributed continuous variables, whereas the Mann–Whitney U test was used for non-normally distributed variables. The interval between the biopsy date and the date of death was computed to calculate overall survival. The Kaplan–Meier estimator was used to compute overall survival rates, whereas the log-rank test was used to assess differences between survival curves. A multivariate analysis was conducted to examine the relationships between overall survival and prognostic parameters. Logistic and Cox regression analyses were performed to examine the associations between survival, survival duration, and potential predictors. A *p*-value of less than 0.05 was considered statistically significant.

During the preparation of this manuscript, the authors used generative AI (Google Gemini 1.5 Pro) solely for spell checking and grammatical refinement. After using this tool, the authors reviewed, edited, and verified the content as necessary and take full responsibility for the final content of the publication.

## 3. Results

### 3.1. Results in the Patient Group Whose Resection Materials Were Evaluated

Ninety-seven patients who were diagnosed with NSCLC based on the evaluation of resection materials and who underwent at least one of the EGFR, ALK, and ROS1 molecular tests were serially included in this study.

#### 3.1.1. Clinical and Histopathological Results

Of the patients, 71 (73.2%) were male and 26 (26.8%) were female. The age range was between 46 and 86 years, with a mean age of 62.52. Forty-five (65.2%) patients had a history of smoking. The distribution by clinical stage was as follows: 38 (39.2%) had early-stage disease (stage 1 and stage 2), 25 (25.8%) had locally advanced disease (stage 3), and 10 (10.3%) had advanced disease (stage 4). Of the 58 patients for whom recurrence data were available, 12 (12.4%) had local recurrence and 37 (38.1%) had distant recurrence. Of the 66 patients for whom metastasis status could be evaluated, 53 (54.6%) had metastatic disease.

Of the 37 patients with early-stage disease, 21 received adjuvant chemotherapy, while none of the EGFR-mutant patients received additional adjuvant EGFR-TKI therapy. In total, 19 of the 21 patients with locally advanced disease received chemoradiotherapy (sequential or concurrent), and four of these EGFR-mutant patients received additional EGFR-TKI therapy. All 45 EGFR-wild-type patients with metastatic disease received systemic chemotherapy as first-line therapy, while all 12 EGFR-mutant patients received EGFR-TKI therapy. Ten patients received first-line therapy, and two received second-line therapy following systemic chemotherapy. Three of the EGFR-TKI therapies included crizotinib, eight included erlotinib, and one included afatinib.

Of the resection materials, three (3.5%) were pneumonectomies, 64 (74.4%) were lobectomies, and 19 (22.1%) were wedge resections. The distribution of tumors according to histological types was as follows: adenocarcinoma in 71 (73.2%) cases and non-adenocarcinoma in 12 (12.4%) cases.

In the histopathological evaluation, the distribution according to tumor grade was as follows: nine (13.6%) were grade 1, 37 (56.1%) were grade 2, and 20 (30.3%) were grade 3. Perineural invasion was observed in four (5.2%) patients, pleural invasion in 35 (47.9%) patients, and lymphovascular space invasion in 51 (66.2%) patients. Lymph node metastases were present in 35 (54.7%) patients. The distribution of patients according to pTN staging was as follows: 17 patients (27%) were pT1, 27 patients (42.9%) were pT2, 15 patients (23.8%) were pT3, four patients (6.3%) were pT4, 35 patients (54.7%) were pN0, 15 patients (23.4%) were pN1, and 14 patients (21.9%) were pN2.

#### 3.1.2. Molecular Results

ALK translocation was detected in three patients (3.7%). ROS1 translocation was detected in two patients (3.1%). EGFR mutations were observed in 23 (24%) patients. ALK and ROS1 translocations were not observed in any patients with EGFR mutations. The distribution of EGFR mutations according to exon regions was as follows: 10 (10.3%) patients had exon 19 deletions, and seven (7.2%) had exon 21 point mutations. Three patients (3.1%) had the T790M mutation in exon 20, which is associated with treatment resistance, in addition to an exon 19 deletion. Two patients (2.1%) had a T790M mutation in exon 20, which is associated with treatment resistance, in addition to an exon 21 point mutation. One patient (1%) had an S768I mutation in exon 20, which is also associated with treatment resistance, in addition to an exon 21 point mutation.

Of the 23 patients with EGFR mutations, 16 (61.5%) were female and seven were male. The relationship between female sex and EGFR mutation was statistically significant (*p* < 0.001). The relationship between sex and the exonic region where the mutation occurred was not significant. The mean age of the patients was 62.5 years, and there was no significant relationship between age and EGFR mutation. A history of smoking was found in 44 patients, two of whom had EGFR mutations. Non-smokers had an EGFR mutation rate of 81.8%, while current smokers had a rate of 4.5%. There was a statistically significant relationship between no smoking history and EGFR mutant status (*p* < 0.001). There were no statistically significant relationships between histological type, predominant histological pattern, tumor grade, lymph node metastasis, lymphovascular invasion, perineural invasion, pleural invasion, and EGFR mutation status and the exonic region. The relationships between TTF-1 and Napsin-A expression, EGFR mutation status, and exonic region were also not significant (the demographic, clinical, and histopathological parameters examined in relation to EGFR mutation status are given in detail in Table 1).

#### 3.1.3. The Association Between Molecular Results and Prognosis

Median overall survival was 60 ± 6.2 months, and median disease-free survival was 28.8 ± 4.5 months. The median follow-up was 46 months. Cumulative one-year, three-year, and five-year overall survival rates were 88%, 54%, and 43%, respectively.

**Figure 3:**
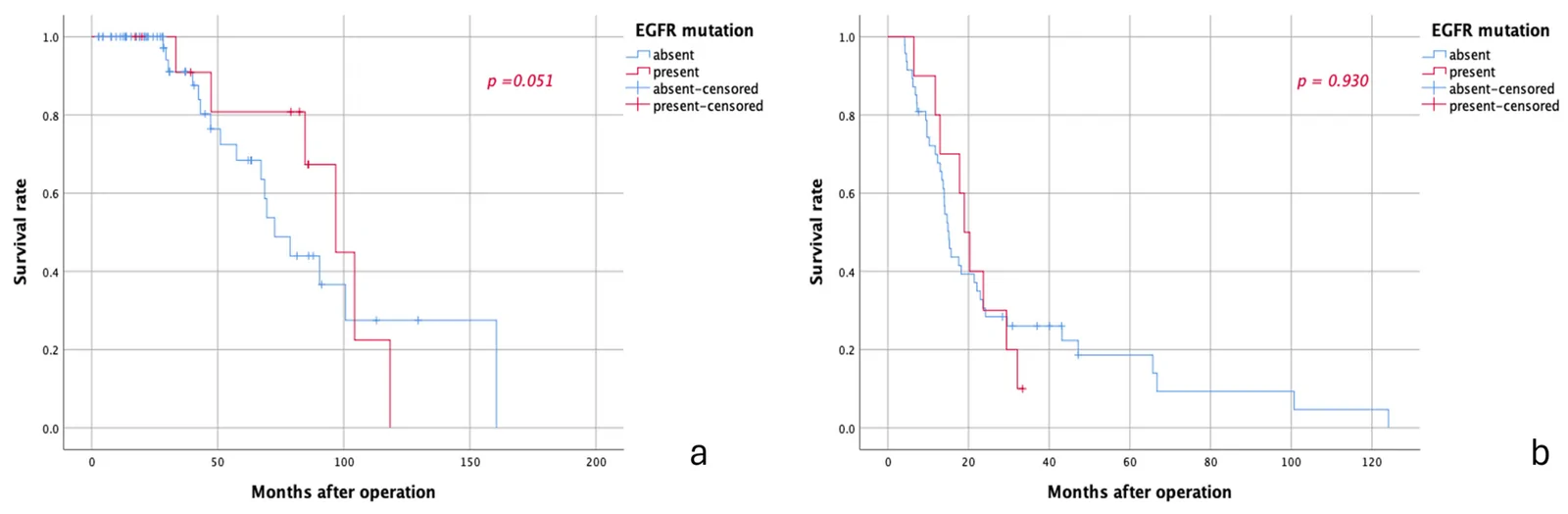
Kaplan–Meier survival curves for patients with and without EGFR mutations: (**a**). median overall survival (OS) times between the EGFR-mutant and EGFR wild-type groups; (**b**). disease-free survival (DFS) times demonstrating the clinical course based on EGFR mutation status (*p*-values were calculated using the log-rank test).

There was no significant association between mortality, recurrence, and metastasis parameters and EGFR mutation, ALK, and ROS1 translocation status. Median overall survival and disease-free survival times in patients with and without EGFR mutations were 81.7 vs. 53.9 months (log rank; *p* = 0.051) and 20.6 vs. 29.8 months (log rank; *p* = 0.93), respectively (Figure 3). The relationships between EGFR mutations and the mutated exon region and median overall survival and disease-free survival times were not statistically significant.

In our multivariate model created with clinical stage, metastasis, and treatment status variables in logistic regression analysis, metastasis was found to be an independent poor prognostic factor for overall survival (OR 0.1, 95% CI: 0.3 to 0.8, *p* = 0.025).

### 3.2. Results in the Patient Group Whose Biopsy Materials Were Evaluated

This study included 141 patients diagnosed with non-small cell lung cancer (NSCLC) based on biopsy material and who underwent at least one EGFR, ALK, or ROS1 molecular test or PD-L1 immunohistochemistry assessment.

#### 3.2.1. Clinical and Histopathological Results

Of the patients, 101 (71.6%) were male and 40 (28.4%) were female. The age range was between 45 and 90, with a mean age of 66.5 years. Sixty-one (56.5%) patients had a history of smoking. The distribution by clinical stage was as follows: 12 (8.5%) early stage (stage 1 and stage 2), 19 (13.5%) locally advanced (stage 3), and 84 (59.6%) advanced stage (stage 4). Ninety-four patients had metastatic disease. Among patients diagnosed at an early stage, one patient received definitive chemoradiotherapy and 10 patients underwent surgery. Relapse occurred in seven of these patients. Six of the patients with locally advanced disease underwent definitive chemoradiotherapy. All of these patients relapsed. Among the patients with metastatic disease, nine with driver mutations received first-line targeted tyrosine kinase inhibitor therapy, while 75 patients without driver mutations received systemic chemotherapy. All patients without driver mutations received systemic chemotherapy regardless of PD-L1 levels. Nineteen of the patients with metastatic disease received nivolumab as immunotherapy at some stage of their treatment. Immunotherapy was administered as second-line therapy in eight patients and as third-line therapy in eight patients.

The distribution by histological type was as follows: adenocarcinoma in 104 (75.4%), squamous cell carcinoma in 14 (10.1%), and NSCLC-not otherwise specified (NOS) in 20 (14.5%).

PD-L1 expression was present in 70 cases (49.6%) and absent in 71 cases (50.4%). PD-L1 expression was less than 1% in 12 cases (14.6%), between 1% and 49% in 40 cases (48.8%), and 50% or greater in 30 cases (36.6%).

The relationships between gender, smoking status, and PD-L1 expression and levels were not statistically significant. The association between PD-L1 expression status and non-adenocarcinoma histological types, particularly squamous cell carcinoma (10.1%) and NSCLC-NOS (14.5%), was statistically significant; PD-L1 positivity was higher in non-adenocarcinoma tumors (70.6%) compared to adenocarcinomas (43.3%; *p* = 0.009). The relationship between PD-L1 expression levels in different histological subtypes was also investigated. A secondary subgroup analysis was performed by dividing the PD-L1-positive biopsy cohort into low-expression (1–49%) and high-expression (50% and above) categories. Among adenocarcinoma cases, 25 (55.6%) showed low expression, while 20 (44.4%) showed high expression. In contrast, in the non-adenocarcinoma group, squamous cell carcinoma cases showed low and high PD-L1 expression rates in three (37.5%) and five (62.5%) cases, respectively, while in NSCLC-NOS cases, 11 (68.8%) showed low expression and five (31.3%) showed high expression. Although patients had non-adenocarcinoma tumors, a comparison of low- and high-tumor-rate-score (TPS) subgroups did not reveal a statistically significant association with histological type, despite a generally higher PD-L1 positivity rate (*p* = 0.324).

The demographic, clinical, and histopathological parameters examined in relation to PD-L1 expression status are detailed in Table 2. The relationships between molecular statuses (EGFR, ALK, and ROS1) and clinicopathological parameters in the biopsy cohort are summarized in Table 3.

#### 3.2.2. Molecular Results

EGFR mutations were present in 17 (12.1%) patients, and BRAF mutations were present in three (2.1%) patients. ALK translocations were present in 17 (12.9%) patients, and ROS-1 translocation was present in one (1.5%) patient. The relationships between EGFR and BRAF mutation status, ALK and ROS-1 translocation status, and PD-L1 expression were not significant (Table 4).

#### 3.2.3. The Association Between Molecular Results and Prognosis

The median overall survival was 16.3 ± 2.1 months, and the median disease-free survival was 5.1 ± 2.1 months. The median follow-up was 20 months. The cumulative one-year, three-year, and five-year overall survival rates were 89%, 69%, and 48%, respectively.

There were statistically significant relationships between mortality rates and male sex (*p* = 0.03), advanced clinical stage (*p* = 0.029), non-adenocarcinoma histological type (*p* = 0.027), and the absence of TTF-1 (*p* = 0.037) and Napsin-A (*p* = 0.026) expression.

**Figure 4:**
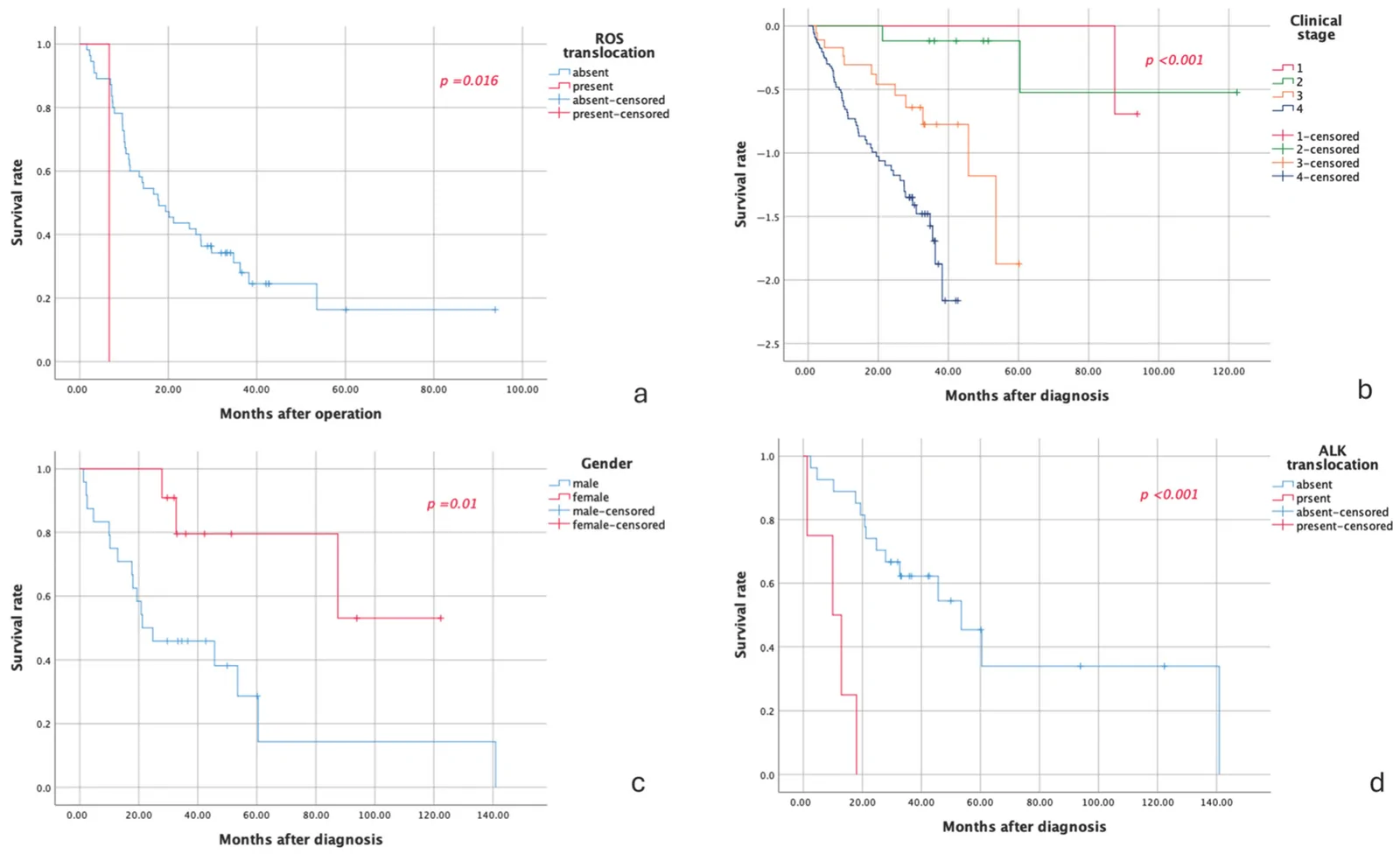
Kaplan–Meier survival curves calculated within the biopsy cohort: (**a**). median overall survival (OS) times in the general patient group with and without ROS1 translocation; (**b**). median OS times across different clinical stages; (**c**). median OS times for women and men in the early-stage and locally advanced-stage groups; (**d**). median OS times in the early-stage and locally advanced-stage groups with and without ALK translocation (*p*-values were calculated using the log-rank test).

Overall survival was significantly lower in patients with ROS-1 translocation compared to the others (6.6 vs. 31.6 months, respectively) (log rank; *p* = 0.016) (Figure 4a). Overall survival was also lower in patients with advanced clinical stage compared with those with early clinical stage (17.4 vs. 90.6 months, respectively) (log rank; *p* < 0.001) and in treated patients compared with untreated patients (12.5 vs. 63.7 months, respectively) (Figure 4b).

In the logistic regression analysis, not receiving immunotherapy was found to be an independent poor prognostic factor for overall survival in our multivariate model created with gender, clinical stage, histological type, TTF-1 and Napsin-A expression status, and treatment status parameters (OR: 0.14, 95% CI: 0.05–0.916, *p* = 0.038). In our multivariate model, which included gender, clinical stage, ROS-1 and ALK translocation status, EGFR mutation status, and therapy status in Cox regression, the presence of ROS-1 translocation was found to be an independent poor prognostic factor for overall survival (HR: 11.2, 95% CI: 1.2–103.9, *p* = 0.033).

The patient series was divided into early-stage, locally advanced-stage, and advanced-stage groups, and survival analyses were re-evaluated independently due to variations in life expectancy and treatment strategies among patients at different clinical stages.

#### 3.2.4. Survival Results in the Advanced-Stage Patient Group

In the group with advanced disease, the median overall survival was 19 ± 2 months. The association between not receiving immunotherapy and the mortality rate was statistically significant (*p* = 0.043).

Overall survival was significantly shorter in patients with ROS-1 translocation compared to those without (6.6 vs. 18.8 months) (log rank; *p* = 0.03) and in patients who did not receive immunotherapy compared to those who did (16.4 vs. 26 months, respectively) (log rank; *p* = 0.029) (Figure 4c).

In the logistic regression analysis, not having received immunotherapy was found to be an independent poor prognostic factor for overall survival in our multivariate model, which included the parameters of having received immunotherapy and BRAF mutation and PD-L1 expression statuses (OR: 0.165, 95% CI: 0.031–0.883, *p* = 0.035). In our multivariate model, we included parameters such as immunotherapy status, BRAF and EGFR mutation status, ALK and ROS-1 translocation status, and PD-L1 expression status in our Cox regression analysis. We found that not having received immunotherapy was an independent poor prognostic factor for overall survival (HR: 0.289, 95% CI: 0.085–0.983, *p* = 0.047).

#### 3.2.5. Survival Results in Early-Stage and Locally Advanced Patient Groups

The median overall survival in the early-stage and locally advanced disease group was 62 ± 11 months. The median disease-free survival was 140 months. The associations between male gender (*p* = 0.007), the absence of Napsin-A expression (*p* = 0.005), and mortality were found to be statistically significant.

Overall survival time was significantly shorter in males than in females (44.3 vs. 94.3 months, respectively) (log rank; *p* = 0.01) and in patients with ALK translocation than in those without (10.5 vs. 70.1 months, respectively) (log rank; *p* < 0.001) (Figure 4d).

In the Cox regression analysis, the absence of ALK translocation was found to be an independent poor prognostic factor for overall survival in our multivariate model created with the parameters of gender, histological type, PD-L1 expression, EGFR mutation, and ALK translocation status (HR: 8.6, 95% CI: 1.9–38, *p* = 0.004).

## 4. Discussion

EGFR tyrosine kinase inhibitor (TKI) therapy plays an important role in non-small cell lung cancer, and significant improvements in patient survival have been achieved with the use of TKIs. EGFR mutation status is known to be the most important determinant of TKI therapy [9,10]. Variations in patient survival, as well as side effects such as acute lung injury and interstitial pneumonia due to gefitinib use, limit the use of TKIs [23]. Therefore, numerous studies have been conducted to enhance our understanding of the molecular, histopathological, and clinical associations that may be significant in selecting patients who are likely to benefit from TKI therapy. Numerous studies have investigated the clinical and demographic features associated with EGFR mutations. These studies indicate that factors such as Asian ethnicity, younger age, female gender, and a lack of smoking history are correlated with the presence of EGFR mutations. Additionally, patients who exhibit these characteristics tend to respond more favorably to TKIs [18,24].

It is known that the presence of micropapillary and solid patterns is associated with a worse prognosis than the presence of lepidic, acinar, and papillary patterns [24,25]. There are some studies showing that EGFR mutations are more common in the papillary and micropapillary subtypes than in the lepidic and solid subtypes [25,26]. However, there are other studies suggesting that EGFR mutations are more common in tumors with a predominant lepidic pattern [27]. In a study investigating the relationships between molecular features and clinical and histopathological parameters in 345 resection specimens diagnosed with lung adenocarcinoma, a significant association was found between EGFR mutations, female sex, and the absence of a smoking history. This study also investigated the relationship between EGFR mutations and tumor grade, clinical stage, lymphovascular space invasion, and pleural invasion parameters, but no significant results were obtained. The same study noted that acinar growth patterns were more common in patients with EGFR mutations, but this relationship was not statistically significant. However, a statistically significant association was found between the absence of a solid growth pattern and EGFR mutation [28].

In our study, similar parameters were evaluated with EGFR mutations, and no significant relationship was found between tumor grade, clinical stage, lymphovascular invasion, and pleural invasion. No statistically significant relationship was observed between the dominant tumor pattern and EGFR mutations. However, similarly to the study results, the acinar pattern rate was higher in the EGFR mutation group (43%), and the solid pattern rate was higher in the non-EGFR mutant group (88%).

A study including an Asian cohort, which assessed 382 resection and biopsy specimens, revealed that the predominant EGFR mutation site was the L858R mutation in exon 21, followed by a deletion in exon 19. No substantial association existed between the EGFR mutation site and gender, smoking history, or histopathological characteristics. Histopathological assessment revealed that adenocarcinoma morphology and TTF-1 expression are associated with EGFR mutations [29]. In our series, exon 19 deletions were the most common. Similarly to the study results, there was no significant association between the exon region where the mutation occurred and gender, smoking history, histological type, or predominant histological pattern.

Some studies reveal no substantial relationship between EGFR mutation status and TTF-1 or Napsin-A expression [30], despite prior research indicating a strong link between the two [31]. According to our data, there is no statistically significant relationship between the EGFR mutation and TTF-1 or Napsin-A expression.

In our study, consistent with the literature, we found a significant association between EGFR mutations, female sex, and non-smoking status. Survival analyses in patients with EGFR mutations receiving TKI therapy revealed no significant association between gender, smoking status, and median overall survival. Our results are inconsistent with studies reporting that female sex and non-smoking status are associated with better treatment outcomes and longer life expectancy in patients with EGFR mutations [18,28]. In our study, where we meticulously divided the data into subgroups to ensure homogeneity in prognostic analyses and evaluated each subgroup separately in terms of survival analysis, this finding stands out as a departure from the literature and deserves further consideration.

The understanding that immune checkpoint inhibitors targeting PD-1 and PD-L1 antibodies, alone or in combination with chemotherapy, provide a significant survival advantage in patients with advanced-stage NSCLC without driver mutations has established this treatment regimen as the standard first-line treatment [32,33].

To determine the patient group that can receive immunotherapy, PD-L1 positivity in tumors and tumor-infiltrating lymphocytes (TILs) is evaluated in the tissue, and PD-L1 testing is routinely performed along with molecular tests for targeted therapies in NSCLC patients [16]. The main antibody clones used for PD-L1 immunohistochemical analysis are 22C3 (DAKO), 28-8 (DAKO), and SP263 (ROCHE Ventana) [34]. Different drugs have varying thresholds for use. For first-line treatment of NSCLC, pembrolizumab requires a PD-L1 level of 1% or higher. In contrast, other immunotherapy agents can be utilized regardless of the PD-L1 level [16].

It is reported that the response rate to treatment in patients receiving immune modulator therapy is approximately 20% [33,35]. PD-L1 expression, along with the expression level determined by immunohistochemistry, is currently the most widely utilized clinical predictive biomarker for selecting patients eligible for immunomodulatory therapy. Numerous studies have been conducted to explore additional clinical and pathological parameters that may influence patient prognosis and correlate with PD-L1 expression. Additionally, research has focused on the relationship between driver mutations and PD-L1 expression status [18,32].

In a meta-analysis demonstrating the relationship between clinical and histopathological features and PD-L1 expression, it was reported that PD-L1 expression was associated with male gender, smoking history, squamous cell carcinoma morphology, high tumor grade, and advanced clinical stage [18]. A meta-analysis examining the relationship between prognosis and PD-L1 reported that increased PD-L1 expression was associated with a poor prognosis [36].

A study conducted on 552 patients using the SP263 antibody clone, which investigated the relationship between PD-L1 expression and driver mutations, reported no significant association between driver mutations and PD-L1 expression. The study also evaluated the relationship between EGFR exon regions and PD-L1 expression but found no statistically significant relationships between them [37]. Similarly, our study found no statistically significant association between PD-L1 expression and driver mutations. There was also no significant association between PD-L1 expression and EGFR exon regions.

A study utilizing the 22C3 antibody clone and involving 1005 patients found that the mean overall survival and disease-free survival times were statistically significantly lower in the PD-L1-positive patient group. Additionally, this study identified a significant association between male gender, smoking history, advanced TNM stage, and PD-L1 expression positivity. It also noted that PD-L1 positivity was statistically significantly higher in the EGFR-mutation-negative patient group, while no association was found between other driver mutations and PD-L1 expression status [38]. In our study, no significant association was found between PD-L1 expression and gender or smoking status. While we observed higher rates of PD-L1 expression in locally advanced and advanced-stage disease, we found no significant relationship between disease stage and PD-L1 expression. The relationship between EGFR mutation and PD-L1 expression was also not statistically significant. Overall survival times in the PD-L1-positive and non-positive groups were 18 months and 14 months, respectively. Although the PD-L1-positive group had a longer overall survival time, there was no significant association between PD-L1 expression status and mean overall survival or disease-free survival.

In another study involving 110 patients and utilizing the SP263 clone, researchers investigated the relationship between PD-L1 expression status and various factors, including gender, smoking, and TTF-1 and Napsin-A expression, as well as clinical stage parameters. They found no significant relationships between these factors and PD-L1 expression. Additionally, the study revealed no significant correlation between EGFR mutation status, ALK and ROS-1 translocations, and PD-L1 expression. In survival analyses, PD-L1 expression status was compared among patients with EGFR mutations, indicating that the relationship between EGFR mutations and PD-L1 expression was associated with longer survival [39]. In our study, no significant association was found between PD-L1 expression and TTF-1 and Napsin-A expression or clinical stage. Similarly, no association was found between PD-L1 expression and EGFR mutation, ALK, or ROS-1 translocation. Although overall survival was quite higher in the group with EGFR mutation and PD-L1 expression (28 months in the group with EGFR mutation and PD-L1 expression vs. 9 months in the group without PD-L1 expression), the difference in survival times was not statistically significant.

The main limitation of this retrospective study was that the cohort did not have all the parameters and molecular test results available. Due to the missing data in the archive records, some subcategory totals in our analyses reflect only the validated available data, and cases with completely missing follow-up data had to be excluded, which limited our final sample size.

In our overall series, EGFR mutations were present in 40 patients (16.8%), ALK translocations were present in 20 patients (8.4%), and ROS-1 translocations and BRAF mutations were present in three patients each (1.2%). The two most frequent mutations were the E746_A750 deletion in exon 19 and the L858R point mutation in exon 20.

In our series, 24% of resection specimens and 12.1% of biopsy material harbored EGFR mutations, and 12.9% of biopsies and 3.7% of resections harbored ALK translocations. These frequencies and the apparent discrepancy between biopsy and resection cohorts may seem, at first glance, to be inconsistent with the recent literature reporting similar stage-independent driver mutation rates. However, our results can be explained by different clinical and epidemiological factors in a real-world setting.

First, our cohort represents a clinically enriched patient population subject to selective referral patterns rather than an unselected universal screening group. In surgical resections, which predominantly comprised localized (Stage I–II) adenocarcinomas, molecular testing was frequently requested selectively for patients with a high pre-test probability (e.g., non-smokers, females, or younger individuals), thereby artificially enriching the resection group for EGFR alterations. Conversely, the biopsy cohort mainly consisted of advanced/metastatic (Stage IV) cases (59.6%) and included non-adenocarcinoma histological subtypes (NSCLC-NOS and squamous cell carcinoma) referred for routine reflex biomarker testing prior to systemic therapy or immunotherapy. In clinical practice, advanced-stage presentation in younger, non-smoking patients with rapid clinical progression frequently triggered targeted ALK FISH testing, resulting in a higher proportion of ALK-positive cases within the biopsy cohort. Because the biopsy and resection materials represent demographically and clinically non-identical patient subsets rather than a single unified cohort tested universally, direct cross-cohort rate comparisons reflect real-world clinical referral biases rather than true stage-dependent biological divergence.

Secondly, regional epidemiological data increasingly confirm that the mutational makeup of non-small cell lung cancer (NSCLC) in Türkiye constitutes a distinct intermediate region showing higher prevalences of driver mutations compared to Western European and North American populations. Multicenter Turkish epidemiological studies and comprehensive institutional pathology series have consistently documented baseline EGFR mutation rates ranging from 18% to 24% in targeted adenocarcinoma cohorts. These findings suggest that geographic, environmental, and potential ethnic differences play a decisive role in driver oncogene distribution and validate the overall higher rates observed in our clinical practice [4,5,40].

No significant associations were found between EGFR mutations and the mutated exon region, histological type, tumor grade, dominant histological pattern, TTF-1 and Napsin-A expression, pleural invasion, perineural invasion, lymph node metastasis, pTNM stage, and clinical stage parameters. However, our study is considered important because it evaluated a larger number of parameters, some of which had not been previously investigated, compared to other studies in the literature.

In our series, survival analyses conducted on EGFR-mutated patient groups receiving tyrosine kinase inhibitors (TKIs) revealed no significant association between gender or smoking status and mean overall survival time. In the immunotherapy patient group, which included those with PD-L1 expression levels below 1%, the mean overall survival time was significantly longer. In both early-stage and locally advanced-stage patient groups, we found that patients with ALK translocation had significantly lower overall survival rates than those reported in the literature and that this was an independent poor prognostic factor. Our findings are particularly valuable because of the large number of patients with ALK translocation in our study. Crucially, it must be emphasized that these early and locally advanced ALK-positive patients underwent standard curative-intent modalities (radical surgery and/or definitive chemoradiotherapy) rather than upfront targeted tyrosine kinase inhibitor (TKI) therapy. This clinical pathway was dictated by the strict regulatory frameworks within our healthcare system, where reimbursement and access to frontline TKIs were rigidly restricted to advanced-stage (Stage IV) or metastatic disease. The lack of early, targeted oncogenic inhibition in this non-metastatic but biologically aggressive subgroup likely accounts for the significantly poorer survival trajectory observed in our cohort. Consequently, these real-world data strongly suggest that expanding TKI access to early and locally advanced settings could critically pivot the therapeutic landscape and improve long-term survival outcomes for ALK-positive patients.

Furthermore, while our current study primarily focused on standalone PD-L1 expression, contemporary thoracic oncology is rapidly evolving toward the utilization of composite biomarker algorithms. Emerging clinical data increasingly suggest that integrating PD-L1 scoring with companion multi-omic metrics, such as tumor mutational burden (TMB) and microsatellite instability (MSI), may provide a far more comprehensive and nuanced prediction of immunotherapy responsiveness. Implementing such multifaceted profiling strategies could potentially resolve the heterogeneous clinical outcomes and therapeutic resistance mechanisms frequently observed across distinct driver-mutant cohorts.

## 5. Conclusions

This study contributes to the literature by providing reliable information on topics with conflicting data, as it presents comprehensive prognostic data in homogeneous subgroups. Targeted therapies have been shown to significantly improve survival rates in patients with NSCLC. However, the existing literature reveals a variety of differing results concerning the factors associated with EGFR mutations that may predict responses to tyrosine kinase inhibitor (TKI) therapy. This variability highlights a lack of consensus in the field and underscores the need for additional data and further studies. Looking ahead, it is believed that immunohistochemical assessments of PD-L1 expression levels—when used not alone but in conjunction with broader genomic biomarkers, such as tumor mutation burden (TMB) and microsatellite instability (MSI), currently being evaluated in the literature—may help in more accurately predicting treatment outcomes. Furthermore, analyses for driver mutations should be conducted for all patients, irrespective of demographic, clinical, or histopathological characteristics or disease stage.

## Figures and Tables

**Table 1:** In patients whose EGFR mutation status was evaluated, the relationships between EGFR mutation status and demographic, clinical, and pathological parameters were examined.

Parameters	EGFR Mutant Type *n* (%)	EGFR Wild Type *n* (%)	*p*
Gender			<0.001
Female	16 (61.5%)	10 (38.5%)	
Male	7 (10%)	63 (90%)	
Smoking history *			<0.001
Presence	2 (4.5%)	42 (95.5%)	
Absence	9 (81.8%)	2 (18.2%)	
Clinical stage *			0.357
Early stage	6 (15.7%)	32 (84.3%)	
Locally advanced stage	5 (20%)	20 (80%)	
Advanced stage	0	9 (100%)	
Histological type *			0.544
Adenocarcinoma	19 (37.2%)	51 (62.8%)	
Non-adenocarcinoma	1 (8.3%)	11 (91.7%)	
p T *			0.103
T1-T2	10 (16.3%)	51 (83.7%)	
T3-T4	1 (8.3%)	11 (91.7%)	
p N *			0.532
N0	6 (17.6%)	28 (82.4%)	
N1	3 (20%)	12 (80%)	
N2	3 (21.4%)	11 (78.6%)	
Tumor grade *			0.089
Grade 1	5 (55.6%)	4 (44.4%)	
Grade 2	10 (27%)	27 (73%)	
Grade 3	3 (16.7%)	16 (84.3%)	
Dominant histological pattern *			
Lepidic pattern	4 (50%)	4 (50%)	0.140
Acinar pattern	10 (31.1%)	22 (68.9%)	0.362
Papillary pattern	2 (25%)	6 (75%)	0.112
Solid pattern	2 (11.7%)	15 (88.3%)	0.078
Micropapillary pattern *			0.574
Presence	3 (30%)	7 (70%)	
Absence	19 (33.3%)	38 (66.7%)	
Lymph node metastasis *			0.351
Presence	6 (24.1%)	29 (75.9%)	
Absence	7 (82.9%)	22 (17.1%)	
Lymphovascular invasion *			0.248
Presence	11 (21.6%)	40 (78.4%)	
Absence	3 (12%)	22 (88%)	
Pleural invasion *			0.144
Presence	9 (25.7%)	26 (74.3%)	
Absence	5 (13.2%)	33 (86.8%)	
Perineural invasion *			0.536
Presence	1 (25%)	3 (75%)	
Absence	12 (16.7%)	60 (83.3%)	
TTF-1 expression *			0.101
Positive	18 (25.4%)	53 (74.6%)	
Negative	1 (6.7%)	14 (93.3%)	
Napsin A expression *			0.228
Positive	12 (24.5%)	37 (75.5%)	
Negative	2 (11.8%)	15 (88.2%)	

* Due to the retrospective nature of this study, certain missing clinical and histopathological data could not be accessed from the archive records.

**Table 2:** Relationships between PD-L1 expression status and demographic, clinical, and pathological parameters in a biopsy material cohort.

		PD-L1 Expression		*p*
		Absence *n* (%)	Presence *n* (%)	
Gender	Male	53 (52.4%)	48 (47.6%)	0.27
	Female	18(45%)	22(55%)	
Smoking history *	Absence	5 (38.4%)	8 (61.6%)	0.343
	Presence	32 (52.4%)	29 (47.6%)	
Clinical stage *				
	Stage 1	2 (100%)	0	0.622
	Stage 2	5 (50%)	5 (50%)	
	Stage 3	5 (26.3%)	14 (73.7%)	
	Stage 4	44 (52.4%)	40 (47.6%)	
Histological type *	Adenocarcinoma	59(56.7%)	45 (43.3%)	0.009
	Non-adenocarcinoma	10 (29.4%)	24 (70.6%)	
TTF-1 expression *	Negative	15 (44.1%)	19 (55.9%)	
	Positive	46 (55.4%)	37 (44.6%)	0.182
Napsin-A expression *	Negative	16 (51.6%)	15 (48.4%)	
	Positive	33 (50%)	33 (50%)	0.528

* Due to the retrospective nature of this study, certain missing clinical and histopathological data could not be accessed from the archive records.

**Table 3:** Relationship between molecular statuses (EGFR, ALK, and ROS1) and clinicopathological parameters in the biopsy cohort.

Parameters	ALK Positive (*n* = 17)	*p*-Value (ALK vs. WT)	ROS1 Positive (*n* = 1) **	EGFR Positive (*n* = 17)	*p*-Value (EGFR vs. WT)
Gender		0.142			0.018
Male	10 (58.8%)		1 (100%)	8 (47.1%)	
Female	7 (41.2%)		0 (0%)	9 (52.9%)	
Smoking history *		1.000			0.008
Absence	3 (42.9%)		0 (0%)	5 (55.6%)	
Presence	4 (57.1%)		1 (100%)	4 (44.4%)	
Clinical stage *		0.364			0.262
Stage 1	0 (0%)		0 (0%)	0 (0%)	
Stage 2	0 (0%)		0 (0%)	3 (21.4%)	
Stage 3	2 (18.2%)		0 (0%)	2 (14.3%)	
Stage 4	9 (81.8%)		1 (100%)	12 (71.4%)	
Histological type *		0.364			0.013
Adenocarcinoma	11 (68.8%)		0 (0%)	17 (100%)	
Non-adenocarcinoma	5 (31.3%)		1 (100%)	0 (0%)	
TTF-1 expression *		0.500			0.164
Negative	3 (25%)		0 (0%)	2 (13.3%)	
Positive	9 (75%)		1 (100%)	13 (86.7%)	
Napsin-A expression *		0.853			0.116
Negative	3 (30%)		0 (0%)	1 (10%)	
Positive	7 (70%)		1 (100%)	9 (90%)	

Abbreviations: WT, Wild-type. * Parameters include incomplete/unrecorded retrospective clinical data; therefore, the subcategory numbers do not exactly equal the total number of positive cases (*n* = 17 for ALK and EGFR). ** Statistical analysis for ROS1 was not performed due to the presence of only one positive case (*n* = 1). It is presented descriptively only.

**Table 4:** Relationships between PD-L1 expression status and molecular outcomes in a biopsy material cohort.

			PD-L1 Expression	
		Absence N (%)	Presence N (%)	*p*
	Negative	60 (52.2%)	55 (47.8%)	
ALK translocation *				0.149
	Positive	6 (35.3%)	11(64.7%)	
	Negative	34 (47.9%)	37 (52.1%)	
BRAF mutation *				0.541
	Positive	1 (33.3%)	2 (66.7%)	
	Negative	58 (47.2%)	65 (52.8%)	
EGFR mutation *				0.059
	Positive	12 (70.6%)	5 (29.4%)	
	Negative	31 (47%)	35 (53%)	
ROS-1 translocation *				0.478
	Positive	1 (100%)	0	

* Analyses were performed based on the molecular analysis results available in archive records. Molecular analysis results are not available for all patients.

## Data Availability

The data supporting the findings of this study are available from the corresponding author upon reasonable request. (The current study are not publicly available due to privacy and ethical restrictions regarding patient clinical information).
